# To: Prognostic significance of gastrointestinal dysfunction in critically ill patients with COVID-19

**DOI:** 10.62675/2965-2774.20250014

**Published:** 2025-04-16

**Authors:** Josef Finsterer, Fulvio Alexandre Scorza, Carla Alessandra Scorza

**Affiliations:** 1 Neurology and Neurophysiology Center Department of Neurology Vienna Austria Department of Neurology, Neurology and Neurophysiology Center - Vienna, Austria.; 2 Universidade Federal de São Paulo Escola Paulista de Medicina São Paulo SP Brazil Escola Paulista de Medicina, Universidade Federal de São Paulo - São Paulo (SP), Brazil.

To the Editor

We were interested to read the article by Lima et al. on a retrospective study of morbidity and mortality associated with gastrointestinal dysfunction (GID) in critically ill COVID-19 patients in hospital and after one year.^(1)^ Gastrointestinal dysfunction was assessed using a predefined 5-point GID score (GIDS).^(1)^ Gastrointestinal dysfunction score was associated with mortality and the need for mechanical ventilation, and in-hospital and 1-year mortality increased with increasing GIDS.^(1)^ It was concluded that GIDS is a predictor of in-hospital and 1-year mortality in COVID-19 patients.^(1)^ The study is noteworthy, but several points should be discussed.

The first problem is the retrospective design of the study.^(2)^ Retrospective studies inherently carry several disadvantages.^(3)^ As they rely on the review of medical records not initially intended for research purposes, data may be incomplete. Selection and recall errors also affect the results, and the reasons for differences in treatment between patients and those lost to follow-up cannot be determined, which can lead to bias.^(2)^

The second point is that 80% of patients had a GIDS score of only 0, meaning that they had either no symptoms or only one of the symptoms listed in table 1.^(1)^ Because of these only mild symptoms or lack of symptoms, it is quite unlikely that GID actually influenced in-hospital or 1-year mortality and that mortality was actually not due to GID. Risk factors for mortality in ventilated intensive care unit (ICU) patients include a body mass index > 32kg/m^2^, respiratory system compliance < 30mL/cmH_2_O, driving pressure > 14cmH_2_O and SOFA score > 5.8 immediately after the initiation of invasive ventilatory support.^(4)^ A biomarker of mortality due to COVID-19 could be serum cortisol.^(5)^

The third point is that the different causes of GID in COVID-19 patients have not been differentiat.^(1)^ Gastrointestinal dysfunction can be due to infections, immunological, vascular, neurological, psychiatric or gastrointestinal diseases.^(3)^ Before GID can be attributed to SARS-CoV-2 infection, all these different causes must be thoroughly ruled out. GID may also be due to an exacerbation of pre-existing GID prior to COVID-19. Since GID is highly dependent on the underlying cause, it would be interesting to know the causes of GID.

The fourth issue is that the causes of mortality were not documented.^(1)^ Mortality after discharge from hospital may be multicausal and not necessarily related to GID.^(6)^ Therefore, the cause of death must be included in the analysis to exclude patients who died from causes other than GID.

The fifth point is that the GID was assessed by the mean value of the GIDS during the first seven days in the ICU, but one of the inclusion criteria was a length of stay in the ICU of > 24 hours.^(1)^ This means that patients with a short ICU stay did not participate in the GIDS. We should know how many of the patients with GIDs were missed because they were discharged before the GIDS could be applied. How many of the patients discharged from the ICU with a length of stay of less than 7 days developed a GID after discharge?

To summarize, this interesting study has limitations that put the results and their interpretation into perspective. Addressing these limitations could strengthen the conclusions and corroborate the study's message. All unanswered questions need to be clarified before readers uncritically accept the study's conclusions. It is unlikely that GID associated with severe COVID-19 is a predictor of mortality or mechanical ventilation.
